# Supplementary material for: Whole-Genome Sequencing of Two Canine Herpesvirus 1 (CaHV-1) Isolates and Clinicopathological Outcomes of Infection in French Bulldog Puppies
Source: Viruses. 2024 Jan 30;16(2):209. doi: 10.3390/v16020209 (PMC10893542; doi:10.3390/v16020209)
Supplement: Supplementary file 1 [file viruses-16-00209-s001.zip › viruses-2807130-supplementary.pdf]

Supplementary Materials

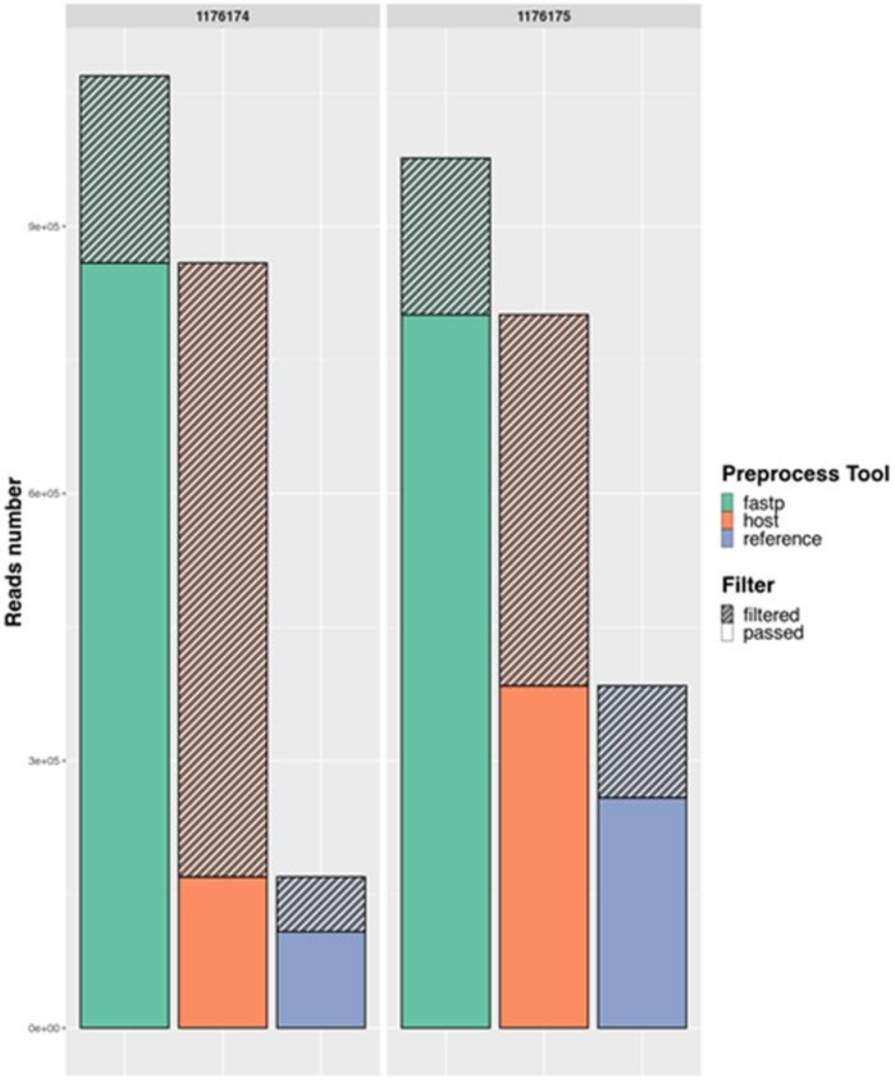

Figure S1. The picture shows the number of reads kept (full background) and discarded (dashed background) during the three steps of preprocessing, referred to fastp quality check, host cleaning, and alignment on CaHV reference genome. Data related to each step, in the same aforementioned order, are depicted with a specific background color, respectively pastel-green, orange and pastel-blue respectively.

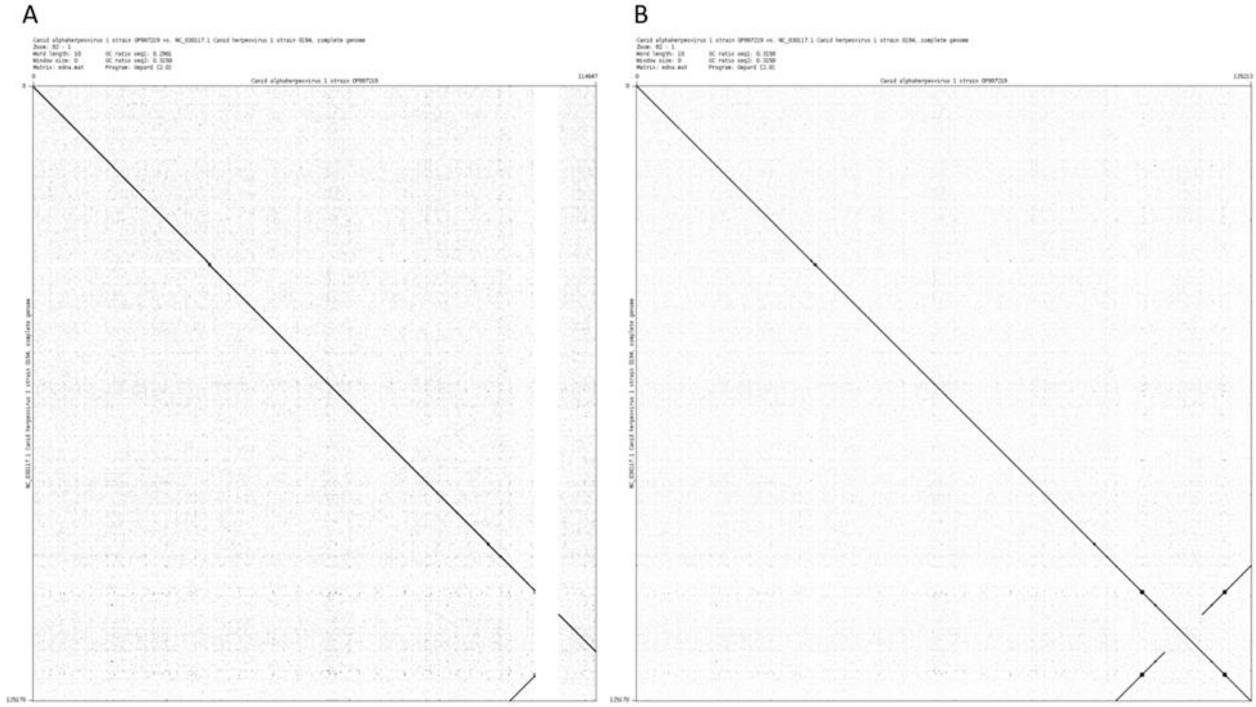

Figure S2. Graphical assemblies evaluation of sample 1176174 (OP997219). The sequences of the two assemblies, the first produced with SPAdes **(A)**, the second with Geneious Prime **(B)**, were compared with the CaHV reference genome (NC_030117) using dot plot made by Gepard (v2.1) [37]. It is clear that the usage of a reference genome as scaffold **(B)** during the assembly process allowed a better characterization of the 3′ terminus repeats, which instead collapsed together in the SPAdes assembled sequence **(A)**.

Table S1. Unique mutations of Sardinian strains (ac.no. OP997219 and OP997220) compared to the reference strain NC_030117

Chrom	Pos	Ref	Alt	Type	Ftype	Strand	Nt_Pos	Aa_Pos	Effect	Locus Tag	Gene	Product
NC_030117	9418	G	A	snp	CDS	+	138/261	46/86	synonymous_variant c.138G>A p.Thr46Thr	A8B60_gp09	UL49A	envelope glycoprotein N
NC_030117	21023	C	T	snp	CDS	-	54/1059	18/352	synonymous_variant c.54G>A p.Gln18Gln	A8B60_gp17	UL42	DNA polymerase processivity subunit
NC_030117	21777	C	T	snp	CDS	+	393/1461	131/486	synonymous_variant c.393C>T p.Tyr131Tyr	A8B60_gp18	UL41	tegument host shutoff protein
NC_030117	35942	C	T	snp	CDS	+	7937/9531	2646/3176	missense_variant c.7937C>T p.Thr2646Ile	A8B60_gp21	UL36	large tegument protein
NC_030117	36197	A	C	snp	Repeat region	+	8192/9531	2731/3176	missense_variant c.8192A>C p.Asn2731Thr	A8B60_gp21	UL36	large tegument protein
NC_030117	36221	A	C	snp	Repeat region	+	8216/9531	2739/3176	missense_variant c.8216A>C p.Asn2739Thr	A8B60_gp21	UL36	large tegument protein
NC_030117	36245	A	C	snp	Repeat region	+	8240/9531	2747/3176	missense_variant c.8240A>C p.Asn2747Thr	A8B60_gp21	UL36	large tegument protein
NC_030117	36269	A	C	snp	Repeat region	+	8264/9531	2755/3176	missense_variant c.8264A>C p.Asn2755Thr	A8B60_gp21	UL36	large tegument protein
NC_030117	36293	A	C	snp	Repeat region	+	8288/9531	2763/3176	missense_variant c.8288A>C p.Asn2763Thr	A8B60_gp21	UL36	large tegument protein
NC_030117	48877	C	T	snp	CDS	+	3306/3594	1102/1197	synonymous_variant c.3306C>Tp.Tyr1102Tyr	A8B60_gp28	UL29	single-stranded DNA-binding protein
NC_030117	58103	C	T	snp	CDS	-	355/1758	119/585	missense_variant c.355G>A p.Asp119Asn	A8B60_gp34	UL25	DNA packaging tegument protein UL25
NC_030117	59548	T	C	snp	CDS	+	240/987	80/328	synonymous_variant c.240T>C p.Leu80Leu	A8B60_gp36	UL23	thymidine kinase
NC_030117	62546	G	A	snp	CDS	+	2143/2394	715/797	missense_variant c.2143G>A p.Ala715Thr	A8B60_gp37	UL22	envelope glycoprotein H
NC_030117	80443	A	ATT	ins	Intergenic region				modifier n.80443_80444insTT			
NC_030117	86423	C	A	snp	CDS	+	2157/2271	719/756	missense_variant c.2157C>A p.Phe719Leu	A8B60_gp51	UL8	helicase-primase subunit
NC_030117	97502	CTT	C	del	Intergenic region				modifier n.97503_97504delTT			
NC_030117	97576	CG	C	del	Intergenic region				modifier n.97577delG			
NC_030117	107448	G	GTCCTCC	ins	CDS	-	278/1176	93/391	disruptive_inframe_insertion c.273_278dupGGAGGA p.Glu91_Glu92dup	A8B60_gp65	US2	virion protein US2
NC_030117	115252	G	GGGAGGA	ins	Intergenic region				modifier n.115252_115253insGGAGGA			
